# Head‐to‐head comparison of TKI and CPI first‐line treatment strategies in advanced renal cell carcinoma—Real‐world data from the German research platform CARAT

**DOI:** 10.1002/ijc.70211

**Published:** 2025-10-29

**Authors:** Peter J. Goebell, Martin Bögemann, Arnd Nusch, Viktor Grünwald, Lothar Müller, Eyck von der Heyde, Uwe M. Martens, Carolin Lennartz, Michaela Koska, Karin Potthoff, Anja Kaiser‐Osterhues, Carsten Grüllich, Michael Staehler, Martina Jänicke, Dominik Marschner, the CARAT Registry Group

**Affiliations:** ^1^ Department of Urology University Hospital Erlangen Erlangen Germany; ^2^ Department of Urology University Hospital Muenster Münster Germany; ^3^ Praxis für Hämatologie und Internistische Onkologie Ratingen Germany; ^4^ Department for Internal Medicine (Tumour Research) and Department for Urology West German Cancer Center, University Hospital Essen Essen Germany; ^5^ Onkologie UnterEms Leer Germany; ^6^ Onkologische Schwerpunktpraxis Hannover Germany; ^7^ Department of Internal Medicine III SLK Clinics Heilbronn Heilbronn Germany; ^8^ Biostatistics iOMEDICO Freiburg im Breisgau Germany; ^9^ Clinical Epidemiology and Health Economics iOMEDICO Freiburg im Breisgau Germany; ^10^ Medical Department iOMEDICO Freiburg im Breisgau Germany; ^11^ Sana Klinikum Hof Hof Germany; ^12^ Department of Urology, Interdisciplinary Center of Renal Tumors Ludwig‐Maximilians‐University of Munich Munich Germany; ^13^ Department of Haematology/Oncology and Palliative Care Klinikum Stuttgart Stuttgart Germany; ^14^ Present address: Department of Hematology, Oncology, and Stem‐Cell Transplantation University Medical Hospital and Faculty of Medicine, Albert‐Ludwigs University Freiburg Germany

**Keywords:** immune checkpoint inhibitor, progression‐free survival, quality of life, registries, renal cell carcinoma, tyrosine kinase inhibitor

## Abstract

The combination of two immune checkpoint inhibitors (CPI) or a CPI with a tyrosine kinase inhibitor (TKI) has expanded the therapeutic options for advanced/metastatic renal cell carcinoma (aRCC) beyond TKI monotherapy. In the absence of head‐to‐head randomized trials comparing these strategies, we estimate their real‐world effectiveness by emulating a hypothetical randomized trial. A total of 936 patients with aRCC from the prospective, observational, multicenter clinical registry CARAT (NCT03374267) starting first‐line treatment after January 15, 2019, were included. Inverse probability of treatment weighting (IPTW) was used to compare first‐line CPI + TKI (*n* = 447), CPI + CPI (*n* = 257), and TKI monotherapy (*n* = 166). Real‐world progression‐free survival (rwPFS), overall survival (OS), and time‐to‐deterioration (TTD) of health‐related quality of life (HRQoL) were analyzed, also stratified by patients' prognostic risk according to the International Metastatic Renal Cell Carcinoma Database Consortium (IMDC) model. IPTW‐adjusted median rwPFS and OS independent of IMDC risk were 12.3 [10.4–15.6] and 29.0 months [25.6–36.3] for TKI + CPI, 8.3 [6.5–10.9] and 21.9 months [16.3–34.5] for CPI + CPI, and 8.5 [6.4–10.0] and 31.7 months [21.0–40.0] for TKI monotherapy. Compared to CPI + TKI, survival tended to be worse for CPI + CPI (rwPFS: hazard ratio (HR) 1.25 [1.00–1.58]; OS: HR 1.25 [0.95, 1.63]). This finding was more pronounced for rwPFS in patients at intermediate risk. Median TTD of HRQoL did not substantially differ between the strategies. Despite the lack of statistically significant HR differences in rwPFS and OS, there was a trend toward superior survival with first‐line CPI + TKI compared to CPI + CPI. TKI monotherapy may remain a viable first‐line treatment option in selected patient populations. Further analyses, preferentially randomized clinical trials, are warranted.

## Graphical abstract

What's new?

The current standard of care for locally advanced or metastatic renal cell carcinoma (aRCC) is either a combination of two immune checkpoint inhibitors (CPI) or a CPI plus a tyrosine kinase inhibitor (TKI). Here, the authors simulate a head‐to‐head trial comparing the real‐world effectiveness of these combination therapies with TKI monotherapy using a large dataset from the prospective, observational, multicenter German clinical registry CARAT. The analysis suggests better survival with CPI + TKI than with two CPIs, and TKI monotherapy remains a viable option, particularly for patients who cannot tolerate immunotherapy.

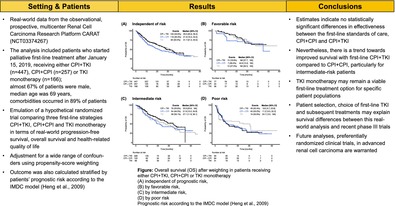

**Abbreviations**
aRCC: advanced or metastatic renal cell carcinoma
AVE: avelumab
AXI: axitinib
BEV: bevacizumab
CAB: cabozantinib
CCI: Charlson comorbidity index
CI: confidence interval
CPI: checkpoint inhibitor
HR: hazard ratio
HRQoL: health‐related quality of life
IFN: interferon‐alpha
IMDC: International Metastatic Renal Cell Carcinoma Database Consortium
IPI: ipilimumab
IPTW: inverse probability of treatment weighting
LEN: lenvatinib
NIV: nivolumab
OS: overall survival
PAZ: pazopanib
PEM: pembrolizumab
PFS: progression‐free survival
PH: proportional hazard
QoL: quality of life
RCT: randomized controlled trial
RMST: restricted mean survival time
rw: real‐world
SMD: standardized mean difference
SOR: sorafenib
StD: standard deviation
SUN: sunitinib
TEM: temsirolimus
TIV: tivozanib
TKI: tyrosine kinase inhibitor
TTD: time to deterioration

## 1 INTRODUCTION

Renal cell carcinoma (RCC) is the most common type of kidney cancer, with a steady increase in incidence worldwide.^1^, ^2^ Approximately one third of patients present with locally advanced or metastatic RCC (aRCC) at diagnosis. Furthermore, every fourth patient receiving treatment for localized disease will relapse.^3^

Systemic therapy, the cornerstone for managing aRCC, has been rapidly evolving over the past decades, with multiple approved strategies and ongoing clinical trials.^4^, ^5^ Until 2005, treatment options for patients with aRCC were largely limited to cytokine‐based therapies, including interferon and interleukin‐2, which demonstrated only modest efficacy.^6^ While vascular endothelial growth factor tyrosine kinase inhibitors (TKI) had previously been the mainstay of first‐line treatment for aRCC, the introduction of immune checkpoint inhibitors (CPI) has led to a paradigm shift in the management of this disease.^4^ The combination of two CPIs or a CPI with a TKI is considered the current standard of care for first‐line treatment of aRCC.^1^, ^2^ These combinations were shown to improve response rate, progression‐free survival (PFS), and/or overall survival (OS) when compared with the TKI sunitinib.^7^, ^8^, ^9^, ^10^, ^11^, ^12^, ^13^, ^14^, ^15^, ^16^ The clinical efficacy of the CPI‐doublet ipilimumab/nivolumab is primarily driven by the International Metastatic Renal Cell Carcinoma Database Consortium (IMDC)^17^ intermediate‐ and poor‐risk categories, while CPI + TKI combinations generally show efficacy across the three IMDC risk groups.^6^ However, there remains a role for TKI monotherapy for patients with favorable‐risk disease according to the IMDC and for those who cannot receive or tolerate immunotherapy.^1^, ^2^ Given the wide range of first‐line treatment options and the absence of head‐to‐head randomized controlled trials (RCT) comparing these different strategies,^4^, ^18^ identifying the optimal treatment for the individual patient—while considering patient and tumor characteristics—poses a challenge for the treating physician.^5^, ^19^ Data from non‐selected patients treated in routine practice are needed to facilitate decision‐making.^5^, ^20^, ^21^

The objective of this analysis is to understand the effectiveness of combination therapy or TKI monotherapy in a real‐world setting. We emulated a head‐to‐head comparison of first‐line CPI + CPI versus CPI + TKI versus TKI monotherapy using a large, prospectively collected dataset from the Renal Cell Carcinoma Research Platform CARAT. After adjusting for a multitude of potential confounders, clinical and patient‐reported outcomes (PRO), that is, real‐world PFS (rwPFS), OS, and time to deterioration (TTD) of health‐related quality of life (HRQoL), were estimated for the different treatment strategies. Estimates were also calculated stratified by patients' prognostic risk according to the IMDC model.

## 2 PATIENTS AND METHODS

### 2.1 Study design and cohort definition

The Renal Cell Carcinoma Research Platform CARAT is an ongoing, prospective, observational, longitudinal, multicenter clinical registry which started in 2017 and continues the Tumor Registry of Advanced Renal Cell Carcinoma (RCC‐Registry).^22^, ^23^, ^24^, ^25^ Eligible patients are ≥18 years of age with a histologically confirmed diagnosis of locally advanced and unresectable or metastatic RCC at the beginning of their palliative first‐line treatment. Inclusion is possible within 12 weeks after the start of first‐line treatment if patients do not participate in the PRO module. All patients must provide written informed consent. By August 2024, a total of 1185 patients were enrolled from 160 study sites, including hospitals and office‐based practices located across Germany. Sites are encouraged to enroll patients consecutively to ensure unselected recruitment and to minimize selection bias. Patients are treated according to the physician's choice based on the patients' individual needs, schedules, and guidelines. No specifications for diagnostic and therapeutical procedures are imposed at any time. Patients are followed for a maximum of 3 years from enrollment or until death, loss to follow‐up, or withdrawal of consent. Data on patients' demographic and clinical (tumor) characteristics as well as prognostic factors and biomarker testing are documented at inclusion. After the initial documentation, data on (sequential) treatments as well as on the course of disease are collected and updated at least every 3 months or at every change of patient or tumor situation. During the follow‐up period, data on additional molecular testing are collected. Outcome parameters assessed as per site standard include tumor response, date(s) of progression(s), and date of death from any cause. Tumor response is documented as the best (clinical) response by the physician and not at previously specified time points according to Response Evaluation Criteria In Solid Tumors (RECIST) criteria. Patients' data are transferred from medical records and comparable information carriers to a secure web‐based electronic case report form (eCRF) by designated site staff and are updated after each follow‐up visit, at any change in therapy, or at least every 3 months. For quality assurance, data plausibility checks are performed, and queries are generated automatically by the eCRF software. Manual checks on data completeness and plausibility are performed regularly to ensure the reliability of the data. For quality assurance reasons, study sites are also contacted for correction or completion of data, if necessary.

For QoL assessment, patients are asked to fill in PRO questionnaires at the time of recruitment before the start of systemic therapy and every 3 months thereafter for a maximum of 2 years. The participation in the PRO module is optional.

Patients can also give informed consent for the leftover tumor samples to be used in translational research projects in the future. Predefined interim analyses are performed annually.

For the present work, we analyzed data on patients who started first‐line therapy after January 15, 2019 (data base cut: December 31, 2023).

### 2.2 Hypothetical target trial emulation and propensity‐score‐weighted time to event analysis

All time to event endpoints were estimated using the Kaplan–Meier method.^26^ rwPFS was defined as the interval between the start of first‐line treatment and the date of progression or death. Patients without an event before the start of second‐line treatment were censored at the start of second‐line treatment. Patients without an event who did not start a second‐line treatment were censored at the last contact date. OS was defined as the interval between the start of first‐line treatment and the date of death from any cause. Patients alive at the data base cut, lost to follow‐up, or alive at the end of their individual observation period were censored at the last contact date. The start of first‐line treatment was defined as the first application of any systemic palliative treatment. The outcome was also calculated stratified by patients' prognostic risk according to the IMDC model.^17^

Comparative effectiveness of different treatment strategies was assessed by emulating a hypothetical randomized trial.^27^ The main components of the target trial are summarized in Table S1. In order to adjust for confounding in the three treatment groups (CPI + TKI, CPI + CPI, and TKI), we used inverse probability of treatment weighting (IPTW) utilizing stabilized propensity‐score weights.^28^, ^29^ For each patient, the propensity score was estimated using logistic regression containing the following variables: age, sex, IMDC risk groups, histology, any comorbidity, Charlson comorbidity index (CCI), metastatic stage, type of metastasis, and number of metastatic sites. The stabilized propensity‐score weights were calculated for the total cohort and recomputed for each IMDC group to allow for a better balance in covariates. To assess the balance in covariates after IPTW, standardized mean differences (SMD) were calculated using the standard deviation of the unadjusted sample according to Zhang et al.^30^ SMDs greater than 0.1 were seen as evidence for imbalance.^31^

Treatment strategies were compared using the hazard ratio (HR) with 95% confidence intervals (CI) based on Cox's proportional hazard (PH) model. In addition, the restricted mean survival time (RMST) has been calculated for *τ* = 45 months. The application of this method is favored in instances where the PH assumption has been violated, but it is also considered a useful secondary measure even when the PH assumption is satisfied.^32^ The RMST can be interpreted as the average survival time during a defined time period ranging from time 0 to a specific follow‐up time point. The simplest implementation of RMST is to take the area under the Kaplan–Meier curve.

### 2.3 Quality of life

Patients who gave informed consent prior to the start of treatment qualified for the PRO survey and were asked to fill in PRO questionnaires at the time of recruitment (baseline) and every 3 months for up to 24 months. PROs were assessed using the validated 19‐item HRQoL questionnaire National Comprehensive Cancer Network (NCCN)‐Functional Assessment of Cancer Therapy (FACT) Functional Assessment of Cancer Therapy–Kidney Symptom Index (FKSI‐19).^33^ Scoring of the questionnaire was performed according to the respective manuals.

TTD was implemented as a measure of longitudinal HRQoL and defined as the time from the start of first‐line treatment to the time of first clinically relevant deterioration of HRQoL or death. The clinically relevant deterioration of each score was defined according to the methodology described by Cella et al., using prespecified threshold values based on score changes from baseline.^34^ In the Kaplan–Meier analysis, an event was defined as the deterioration of a prespecified threshold, as described above, or death; patients without such an event were censored at the time of completion of their last questionnaire. All patients who had completed their baseline and ≥1 further questionnaire were included in the TTD analysis.

### 2.4 Statistical analysis

All statistical analyses and data visualization were performed using R version 4.3.2 (2023‐10‐31) “Eye Holes” (Platform: x86_64‐pc‐linux‐gnu [64‐bit]). Propensity‐score weighting was performed using the WeightIt package, survival analyses were performed using the survival and survminer packages, and RMST was computed using the RMST package for R.

## 3 RESULTS

### 3.1 Cohort description, baseline characteristics and treatment strategies

**FIGURE 1:**
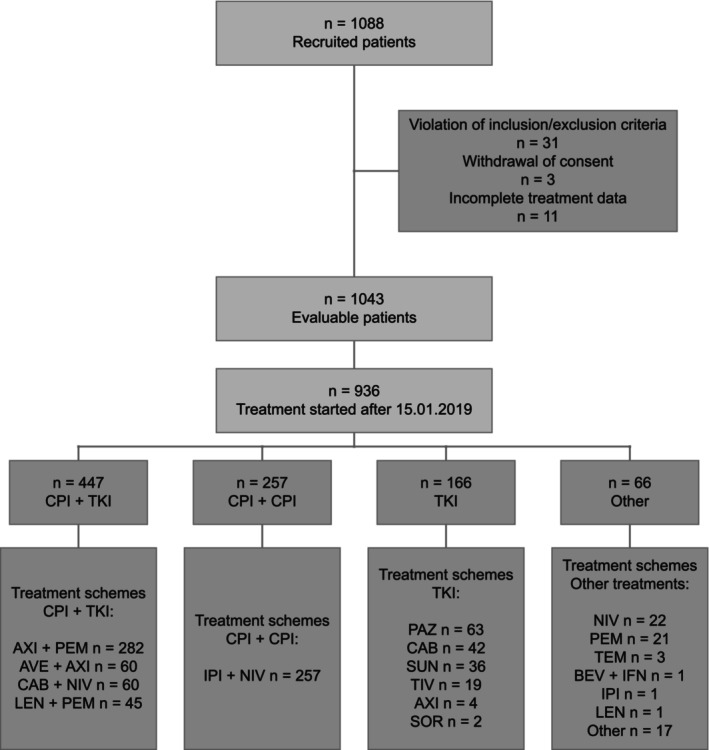
Flow chart. Flow chart of all patients included in this analysis, starting from the total number of patients with advanced renal cell carcinoma recruited into the CARAT registry from December 2017 until December 2023. Our emulation of a target trial is based on those patients who received either checkpoint inhibitor (CPI) + tyrosine kinase inhibitor (TKI) (*n* = 447), CPI + CPI (*n* = 257) or TKI monotherapy (*n* = 166) and started first‐line treatment after January 15, 2019. AVE, avelumab; AXI, axitinib; BEV, bevacizumab; CAB, cabozantinib; IFN, interferon‐alpha; IPI, ipilimumab; LEN, lenvatinib; NIV, nivolumab; PAZ, pazopanib; PEM, pembrolizumab; SOR, sorafenib; SUN, sunitinib; TEM, temsirolimus; TIV, tivozanib.

At the database cut for this analysis (December 31, 2023), a total of 1088 patients with aRCC had been recruited by 145 sites. The present analysis is based on 936 patients who started first‐line treatment after January 15, 2019, with 447 patients (48%) receiving a combination of CPI and TKI, 257 (27%) a combination of two CPIs, 166 (18%) a TKI monotherapy, and 66 (7%) another regimen as first‐line treatment (Figure 1).

Figure 1 also provides details on the most frequently used first‐line regimens. The majority of patients who were treated with CPI + TKI (*n* = 447) received pembrolizumab plus axitinib (*n* = 282, 63%), followed by avelumab plus axitinib and nivolumab plus cabozantinib (each *n* = 60, 13%). A total of 45 patients (10%) were treated with pembrolizumab combined with lenvatinib. All patients with CPI + CPI (*n* = 257) received a combination of ipilimumab and nivolumab. Thirty‐eight percent of patients with TKI monotherapy (*n* = 166) received pazopanib (*n* = 63), followed by cabozantinib (*n* = 42, 25%) and sunitinib (*n* = 36, 22%). The other remaining treatment regimens mainly comprised CPI monotherapy (*n* = 44, 67%; Figure 1).

Baseline characteristics of patients before IPTW are presented in Table 1. Sixty‐seven percent of patients (*n* = 623) were male; the majority (*n* = 829; 89%) presented with comorbidities. Patients receiving CPI + CPI were younger (median age of 65 years) and less frequently of favorable IMDC risk (*n* = 24, 9%) than the patients treated with CPI + TKI or TKI alone.

**TABLE 1:** Baseline characteristics (unadjusted, before inverse probability of treatment weighting ).

Characteristic	CPI + TKI	CPI + CPI	TKI	Other	Total
At start of first‐line treatment	*n* = 447	*n* = 257	*n* = 166	*n* = 66	*n* = 936
Age (years)					
Mean (±StD)	68.08 (±10.30)	65.37 (±10.88)	72.61 (±10.73)	70.50 (±11.12)	68.31 (±10.86)
Median (min–max)	69.40 (34.1–88.9)	64.60 (21.6–90.4)	74.66 (40.1–94.2)	70.94 (43.5–89.7)	69.20 (21.6–94.2)
Sex					
Female	151 (33.8%)	84 (32.7%)	59 (35.5%)	19 (28.8%)	313 (33.4%)
Male	296 (66.2%)	173 (67.3%)	107 (64.5%)	47 (71.2%)	623 (66.6%)
Any comorbidity					
Yes	392 (87.7%)	220 (85.6%)	157 (94.6%)	60 (90.9%)	829 (88.6%)
No	55 (12.3%)	37 (14.4%)	9 (5.4%)	6 (9.1%)	107 (11.4%)
CCI^a^					
0	279 (62.4%)	166 (64.6%)	91 (54.8%)	35 (53.0%)	571 (61.0%)
≥1	168 (37.6%)	91 (35.4%)	75 (45.2%)	31 (47.0%)	365 (39.0%)
Histology					
Clear cell	351 (78.5%)	195 (75.9%)	127 (76.5%)	41 (62.1%)	714 (76.3%)
Non‐clear cell	95 (21.3%)	62 (24.1%)	39 (23.5%)	25 (37.9%)	221 (23.6%)
Missing	1 (0.2%)	0 (0.0%)	0 (0.0%)	0 (0.0%)	1 (0.1%)
Metastases at diagnosis					
M0	172 (38.5%)	85 (33.1%)	72 (43.4%)	23 (34.8%)	352 (37.6%)
M1	214 (47.9%)	139 (54.1%)	60 (36.1%)	28 (42.4%)	441 (47.1%)
MX	58 (13.0%)	33 (12.8%)	34 (20.5%)	15 (22.7%)	140 (15.0%)
Missing	3 (0.7%)	0 (0.0%)	0 (0.0%)	0 (0.0%)	3 (0.3%)
Number of metastatic sites					
0	29 (6.5%)	12 (4.7%)	11 (6.6%)	11 (16.7%)	63 (6.7%)
1	155 (34.7%)	86 (33.5%)	56 (33.7%)	22 (33.3%)	319 (34.1%)
≥2	263 (58.8%)	159 (61.9%)	99 (59.6%)	33 (50.0%)	554 (59.2%)
Selected metastatic locations^b^					
Liver	70 (15.7%)	47 (18.3%)	24 (14.5%)	9 (13.6%)	150 (16.0%)
Lung	256 (57.3%)	173 (67.3%)	104 (62.7%)	37 (56.1%)	570 (60.9%)
Bones	134 (30.0%)	81 (31.5%)	52 (31.3%)	12 (18.2%)	279 (29.8%)
Brain	29 (6.5%)	10 (3.9%)	6 (3.6%)	5 (7.6%)	50 (5.3%)
Pancreas	26 (5.8%)	15 (5.8%)	16 (9.6%)	3 (4.5%)	60 (6.4%)
Clinical IMDC					
Favorable risk	77 (17.2%)	24 (9.3%)	43 (25.9%)	7 (10.6%)	151 (16.1%)
Intermediate risk	225 (50.3%)	123 (47.9%)	65 (39.2%)	27 (40.9%)	440 (47.0%)
Poor risk	95 (21.3%)	85 (33.1%)	29 (17.5%)	19 (28.8%)	228 (24.4%)
Missing	50 (11.2%)	25 (9.7%)	29 (17.5%)	13 (19.7%)	117 (12.5%)

Abbreviations: CCI, Charlson comorbidity index; CPI, checkpoint inhibitor; IMDC, International Metastatic Renal Cell Carcinoma Database Consortium; MX, metastasis cannot be assessed; StD, standard deviation; TKI, tyrosine kinase inhibitor.

^a^ Charlson comorbidity index (CCI) according to Quan et al.^35^

^b^ Multiple answers possible, that is, patients can have more than mentioned metastases (e.g., bone and liver metastases); data collected from 8 weeks before to 4 weeks after start of treatment.

Table 2 presents data on higher‐line treatment. Of all patients included (*n* = 936), first‐line therapy was ongoing for 349 patients (37%) at the time of database cut, while 332 patients (36%) received second‐line treatment. Of all patients with first‐line TKI monotherapy receiving a second‐line treatment (*n* = 91, 55%), the majority (*n* = 70, 77%) were treated with a CPI in second‐line, including 63 patients (70%) with CPI monotherapy. Overall, a total of 210 patients (22%) had died prior to second‐line treatment, and 45 patients (5%) were lost to follow‐up.

**TABLE 2:** Higher‐line treatment (unadjusted, before inverse probability of treatment weighting).

	CPI + TKI *n* = 447	CPI + CPI *n* = 257	TKI *n* = 166	Other *n* = 66	Total *n* = 936
Patients with higher‐line treatment					
Second line	130 (29.1%)	94 (36.6%)	91 (54.8%)	17 (25.8%)	332 (35.5%)
Third line	39 (8.7%)	25 (9.7%)	40 (24.1%)	3 (4.5%)	107 (11.4%)
Fourth line	7 (1.6%)	7 (2.7%)	14 (8.4%)	1 (1.5%)	29 (3.1%)
Patients with second‐line treatment					
TKI	101 (22.6%)	77 (30.0%)	17 (10.2%)	9 (13.6%)	204 (21.8%)
CPI + CPI	4 (0.9%)	1 (0.4%)	4 (2.4%)	0 (0.0%)	9 (1.0%)
CPI + TKI	3 (0.7%)	2 (0.8%)	3 (1.8%)	2 (3.0%)	10 (1.1%)
CPI monotherapy	9 (2.0%)	6 (2.3%)	63 (38.0%)	4 (6.1%)	82 (8.8%)
Other (no TKI, no CPI)	13 (2.9%)	8 (3.1%)	4 (2.4%)	2 (3.0%)	27 (2.9%)
Potential for second line^a^	203 (45.4%)	85 (33.1%)	30 (18.1%)	31 (47.0%)	349 (37.3%)
LTFU prior to second line^b^	24 (5.4%)	10 (3.9%)	8 (4.8%)	3 (4.5%)	45 (4.8%)
Patient died prior to second line^b^	90 (20.1%)	68 (26.5%)	37 (22.3%)	15 (22.7%)	210 (22.4%)
Exposition to TKI and CPI overall					
CPI + TKI (any line)	3 (0.7%)	3 (1.2%)	3 (1.8%)	2 (3.0%)	11 (1.2%)
CPI + CPI (any line)	8 (1.8%)	1 (0.4%)	7 (4.2%)	0 (0.0%)	16 (1.7%)
CPI monotherapy (any line)	15 (3.4%)	8 (3.1%)	70 (42.2%)	5 (7.6%)	98 (10.5%)
TKI monotherapy (any line)	105 (23.5%)	80 (31.1%)	45 (27.1%)	11 (16.7%)	241 (25.7%)

Abbreviations: CPI, checkpoint inhibitor; LTFU, lost to follow‐up; TKI, tyrosine kinase inhibitor.

^a^ Potential: patients whose line of treatment is ongoing or for whom data on a new line of treatment have not yet been documented could still have the option of receiving or not receiving second‐line therapy during the course of the project.

^b^ Based on reasons for the end of the study.

### 3.2 Propensity‐score weighting

In order to reduce selection bias, IPTW was utilized (see Section 2). Patients' baseline characteristics both before and after weighting are presented in Table S2. Balance is determined by an adjusted SMD <0.1. Since all covariates are below 0.1 (Figure S1 and Table 2), SMDs before and after weighting demonstrate a satisfactory balance and thus, the treatment groups were deemed to be comparable for the variables considered.

### 3.3 Response to treatment and clinical outcome

**FIGURE 2:**
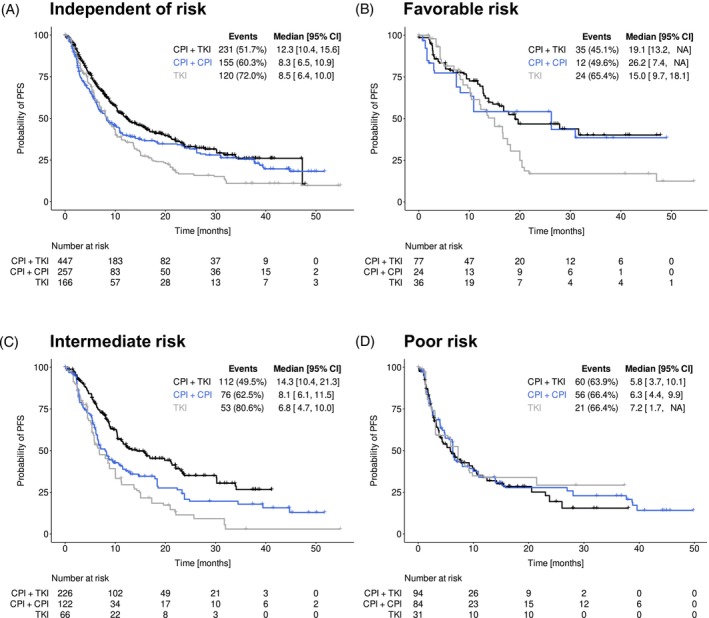
Progression‐free survival (PFS) (adjusted, after inverse probability of treatment weighting). PFS after weighting in patients receiving either checkpoint inhibitor (CPI) + tyrosine kinase inhibitor (TKI), CPI + CPI or TKI monotherapy independent of prognostic risk (A), by favorable risk (B), intermediate risk (C) and poor risk (D) according to the International Metastatic Renal Cell Carcinoma Database Consortium (IMDC) model.^17^ Numbers at risk refer to the sum of weights of the respective patients at risk for a given time point. Due to rounding and missing values for IMDC risk classification, the weights of the three risk groups may not exactly add up to the sum of weights calculated for the cohorts, as shown in (A). CI, confidence interval; NA, not available/not reached.

**FIGURE 3:**
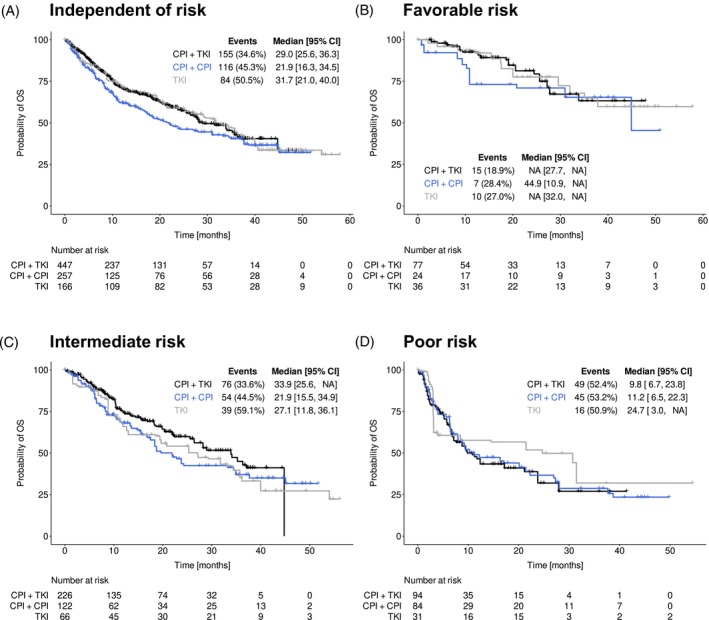
Overall survival (OS) (adjusted, after inverse probability of treatment weighting). OS after weighting in patients receiving either checkpoint inhibitor (CPI) + tyrosine kinase inhibitor (TKI), CPI + CPI or TKI monotherapy independent of prognostic risk (A), by favorable risk (B), intermediate risk (C) and poor risk (D) according to the International Metastatic Renal Cell Carcinoma Database Consortium (IMDC) model.^17^ Numbers at risk refer to the sum of weights of the respective patients at risk for a given time point. Due to rounding and missing values for IMDC risk classification, the weights of the three risk groups may not exactly add up to the sum of weights calculated for the cohorts, as shown in (A). CI, confidence interval; NA, not available/not reached.

Duration of first‐line treatment, reasons for end of treatment, patients' response to treatment and their clinical outcome are shown in Table 3. The Kaplan–Meier curves for PFS and OS after weighting are presented in Figures 2 and 3, respectively.

**TABLE 3:** Response to treatment and clinical outcome of patients.

Characteristics at start of first‐line treatment^a^	CPI + TKI	CPI + CPI	TKI
	*n* = 447	*n* = 257	*n* = 166
Patients with completed first‐line treatment	285 (63.8%)	209 (81.3%)	145 (87.3%)
Treatment duration in months, median (25%–75% quartile)	6.0 (2.8–11.1)	4.1 (1.4–10.2)	5.1 (2.4–12.4)
Reason for end of treatment			
Progression	154 (34.5%)	105 (40.9%)	78 (47.0%)
Toxicity	40 (8.9%)	36 (14.0%)	29 (17.5%)
Patient died	37 (8.3%)	19 (7.4%)	11 (6.6%)
Patient lost to follow‐up	12 (2.7%)	7 (2.7%)	5 (3.0%)
According to treatment plan/guidelines	4 (0.9%)	9 (3.5%)	3 (1.8%)
Other	37 (8.3%)	30 (11.7%)	19 (11.4%)
Missing	1 (0.2%)	3 (1.2%)	0 (0.0%)
Ongoing treatment	162 (36.2%)	48 (18.7%)	21 (12.7%)
Registry best response^b^			
CR	19 (4.3%)	14 (5.4%)	5 (3.0%)
PR	94 (21.0%)	41 (16.0%)	36 (21.7%)
SD	89 (19.9%)	53 (20.6%)	43 (25.9%)
PD	59 (13.2%)	58 (22.6%)	33 (19.9%)
Unknown to site	65 (14.5%)	49 (19.1%)	34 (20.5%)
Missing/ongoing treatment	121 (27.1%)	42 (16.3%)	15 (9.0%)
Real‐world progression‐free survival			
Events	231 (51.7%)	155 (60.3%)	120 (72.0%)
rwPFS in months, median (95% CI)	12.3 (10.4–15.6)	8.3 (6.5, 10.9)	8.5 (6.4–10.0)
HR (CPI + CPI vs. CPI + TKI), estimate (95% CI)		1.25 (1.00–1.58)	
HR (TKI vs. CPI + TKI), estimate (95% CI)		1.51 (1.19–1.91)	
HR (TKI vs. CPI + CPI), estimate (95% CI)		1.20 (0.91–1.58)	
RMST Dif (CPI + TKI – CPI + CPI), estimate (95% CI); *p*‐value		2.49 (−0.82, 5.81); .141	
RMST Dif (CPI + TKI – TKI), estimate (95% CI); *p*‐value		6.22 (2.92, 9.52); <.001^c^	
RMST Dif (CPI + CPI – TKI), estimate (95% CI); *p*‐value		3.73 (−0.06, 7.52); .054	
Overall survival (OS)			
Events	155 (34.6%)	116 (45.3%)	84 (50.5%)
OS in months, median (95% CI)	29.0 (25.6,36.3)	21.9 (16.3, 34.5)	31.7 (21.0, 40.0)
HR (CPI + CPI vs. CPI + TKI), estimate (95% CI)		1.25 (0.95–1.63)	
HR (TKI vs. CPI + TKI), estimate (95% CI)		1.04 (0.77–1.41)	
HR (TKI vs. CPI + CPI), estimate (95% CI)		0.83 (0.60–1.16)	
RMST Dif (CPI + TKI – CPI + CPI), estimate (95% CI); *p*‐value		2.97 (−0.45, 6.40); .089	
RMST Dif (CPI + TKI – TKI), estimate (95% CI); *p*‐value		0.09 (−3.81, 3.98); .966	
RMST Dif (CPI + CPI – TKI), estimate (95% CI); *p*‐value		−2.89 (−7.24, 1.46); .193	

*Note*: Data are *n* (%), unless otherwise indicated. Some percentages might not add up to 100% due to rounding.

Abbreviations: CI, confidence interval; CPI, checkpoint inhibitor; CR, complete response; HR, hazard ratio; PD, progressive disease; PR, partial response; RMST Dif, difference between restricted mean survival estimates with Kaplan–Meier; rwPFS, real‐world progression‐free survival; SD, stable disease; TKI, tyrosine kinase inhibitor.

^a^ Unless otherwise indicated.

^b^ There are no specifications as to the timing, frequency or criteria of tumor assessment, thus registry response data should be considered as the best clinical approximation and might not be identical to the response determined in clinical trials.

^c^ Statistically significant.

At database cut, a total of 64%, 81%, and 87% of patients with CPI + TKI, CPI + CPI, and TKI strategy, respectively, were documented with completed first‐line treatments. The median duration of first‐line treatment was 4.1 months for CPI + CPI, 5.1 months for TKI, and 6.0 months for CPI + TKI. The most common reason for end of treatment was disease progression, followed by toxicity (Table 3).

The Kaplan–Meier estimates for rwPFS after weighting are presented in Figure 2A–D. A total of 52%, 60%, and 72% of patients receiving a CPI + TKI, CPI + CPI, and TKI strategy, respectively, experienced disease progression or death following first‐line treatment (Figure 2A, Table 3). Patients censored for weighted rwPFS (*n* = 215/102/47 for the CPI + TKI, CPI + CPI, and TKI groups, respectively) were most often censored at the last documented contact because first‐line treatment was ongoing (*n* = 165/62/17), followed by censoring at the start of second‐line treatment without prior progression (*n* = 16/15/17), or at the date of last contact prior to loss to follow‐up (*n* = 22/10/7). Very few patients were censored at last contact at the end of the 3‐year observation period (8/14/5) or at the last contact because documentation ended for unknown reasons (*n* = 4/1/1). IPTW‐adjusted median rwPFS independent of IMDC risk was 10.2 months [8.8–11.5] for the overall cohort irrespective of the type of treatment, 12.3 months [10.4–15.6] for TKI + CPI, 8.3 months [6.5–10.9] for CPI + CPI, and 8.5 months [6.4–10.0] for TKI monotherapy. In comparison to TKI + CPI, the corresponding HRs were higher for CPI + CPI (1.25 [1.00–1.58]) and TKI (1.51 [1.19–1.91]), the RMST differences statistically significant for CPI + TKI–TKI: 6.22 [2.92–9.52], *p* < .001 (Table 3). Square brackets denote 95% CIs. This observation was even more pronounced for patients at intermediate IMDC risk (Figure 2C): compared to TKI + CPI, the corresponding HRs/RMST differences were 1.58 [1.16–2.16]/4.74 [1.36–8.11], *p* = .006 for CPI + CPI and 2.16 [1.56–2.99]/7.85 [4.52–11.18], *p* < .001 for TKI. In favorable‐ and poor‐risk patients, there was no significant difference in outcome among the three strategies, with the median PFS not yet reached in some cases (Figure 2B,D).

The Kaplan–Meier estimates for OS after weighting are presented in Figure 3A–D. A total of 35%, 45%, and 51% of patients with a CPI + TKI, CPI + CPI, and TKI strategy, respectively, had died at the time of data cut (Figure 3A, Table 3). Patients censored for weighted OS (*n* = 292/140/83) for the CPI + TKI, CPI + CPI, and TKI groups, respectively, were most often censored at the last documented contact because first‐line treatment was ongoing (*n* = 226/94/41), followed by censoring at the date of last contact prior to loss to follow‐up (*n* = 45/22/17), or at the last contact at the end of the 3‐year observation period (16/21/22). Few patients were censored because documentation ended for unknown reasons (*n* = 5/3/3). IPTW‐adjusted median OS independent of IMDC risk was 29.2 months [24.5–34.9] for the overall cohort irrespective of the type of treatment, 29.0 months [25.6–36.3] for TKI + CPI, 21.9 months [16.3–34.5] for CPI + CPI, and 31.7 months [21.0–40.0] for TKI. In comparison to TKI + CPI, the corresponding HR was higher for CPI + CPI (1.25 [0.95–1.63]) (Table 3). Compared to CPI + CPI, the corresponding HR was slightly lower for TKI (0.83 [0.60–1.16]) (Table 3). Square brackets denote 95% CIs. When stratified by patients' prognostic risk according to the IMDC, there was no significant difference in survival among the three strategies, with the median OS not yet reached in most cases (Figure 3B–D).

### 3.4 Patient‐reported outcomes

Four hundred and forty‐seven patients qualified and gave informed consent for the PRO survey (48% of all patients). The PRO questionnaire return rate of these patients was: 92% (*n* = 413) at baseline, 73% (*n* = 325) at 3 months, 62% (*n* = 275) at 6 months, 57% (*n* = 255) at 9 months, 49% (*n* = 219) at 12 months, 43% (*n* = 190) at 15 months, 37% (*n* = 164) at 18 months, 31% (*n* = 138) at 21 months, and 27% (*n* = 119) at 24 months.

Figure S2 displays the Kaplan–Meier estimates for TTD of HRQoL after weighting. Median TTD of HRQoL did not substantially differ between the three first‐line strategies.

When stratified by patients' prognostic risk according to the IMDC, there was no difference in TTD among the three strategies (data not shown).

## 4 DISCUSSION

The current first‐line standards of care for patients with advanced renal cell carcinoma have not been compared head‐to‐head in RCTs. To the best of our knowledge, this is the first emulated hypothetical randomized trial comparing TKI and CPI first‐line treatment strategies based on a large prospectively collected real‐world cohort. After adjusting for a large set of potential confounders, the results of this analysis indicate no statistically significant HR differences in rwPFS and OS between the treatment strategies. However, there was a tendency toward superior survival with first‐line CPI + TKI compared to CPI + CPI, which was more pronounced for rwPFS in patients with intermediate IMDC risk. TKI monotherapy may also remain a viable option for first‐line treatment.

Median PFS and OS of the first‐line CPI doublet nivolumab/ipilimumab were reported to be 10.3 and 41.6 months, respectively, across clinical trials.^3^, ^9^ In the (pivotal) RCTs, median PFS of first‐line CPI + TKI strategies ranged from 13.5 to 23.6 months, median OS from 39.3 to 46.5 months.^3^, ^7^, ^8^, ^10^, ^12^, ^14^, ^15^ CPI‐TKI combinations have been hypothesized to provide enhanced benefit through complementary mechanisms of action in aRCC when compared to immunotherapy alone.^13^ Despite the absence of statistically significant HR differences, a trend toward improved rwPFS and OS with first‐line CPI + TKI compared to CPI + CPI was evident in our emulated target trial (median rwPFS of 12.3 vs. 8.3 months, HR 1.25 [1.00, 1.5]; median OS of 29.0 vs. 21.9 months, HR 1.25 [0.95, 1.63]). This effect was even more pronounced for rwPFS in patients at intermediate IMDC risk. These findings complement those from—partly unadjusted—retrospective real‐world analyses reporting no survival difference or (a trend toward) improved survival with CPI + TKI combinations.^19^, ^36^, ^37^, ^38^, ^39^ In a retrospective, IPTW‐adjusted comparative analysis using health record‐derived real‐world data, survival in the overall cohort tended to be superior for first‐line axitinib/pembrolizumab compared to ipilimumab/nivolumab, with a median PFS of 10.6 versus 6.9 months and a median OS of 28.9 versus 24.3 months.^37^ Results of the retrospective ARON‐1 study indicate that only patients at intermediate IMDC risk benefit more from CPI + TKI strategies than from CPI doublets, which may be associated with the biological background of different IMDC groups.^19^

CPI + CPI and CPI + TKI combination therapies have demonstrated an OS benefit,^7^, ^8^, ^9^, ^12^, ^16^ or trend,^14^ compared to the TKI sunitinib in the intention‐to‐treat population within classical RCT collections. The results of this emulated target trial indicate comparable OS following CPI‐based combination strategies and TKI monotherapy in first‐line. This apparent discrepancy, including the lower absolute survival observed in our data compared to that observed in clinical trials, may be explained by several factors:

*First*, patients treated in routine practice differ from those included in clinical trials, which may affect the absolute length of survival.^22^, ^25^, ^40^ Patients selected for clinical trials generally present with more favorable baseline characteristics (younger age, fewer comorbidities, and better IMDC score) than the general real‐world patient population. In contrast to the pivotal trials on CPI‐based strategies, patients included in this analysis were older (69 vs. 61–64 years), less likely to be male (67% vs. 71%–78%), less frequently of favorable (16% vs. 21%–35%), and more frequently of poor (24% vs. 9%–21%) prognostic risk according to the IMDC.^7^, ^8^, ^11^, ^12^, ^13^

*Second*, all CPI‐based combination therapies were compared with sunitinib in the pivotal first‐line trials.^7^, ^11^, ^12^, ^16^, ^41^ In our analysis, only 22% of patients in the TKI group had received sunitinib, while the majority were treated with either pazopanib (38%) or cabozantinib (25%) in first‐line. In a recent Cochrane systematic review with network meta‐analyses on first‐line therapy in advanced RCC, the median PFS with sunitinib across 19 RCTs was 9.2 months (range: 5.6–13.2), median OS 28.7 months (16.4–37.8).^3^ For pazopanib and cabozantinib, the median PFS was reported to be 8.8 months (6.8–11.3) and 17 months (12.1–24.9), while the median OS was 31.5 months (21.7–44.8) and 34.2 (17.5–66.7) months, respectively.^3^ This is comparable to our real‐world data, which revealed a median PFS and OS of 8.5 and 31.7 months, respectively, for TKI monotherapy and to those from two retrospective studies.^42^, ^43^ Those studies reported a median PFS of 9.1 months^43^ and median OS values of 26.9^42^ and 29.4 months,^43^ respectively, and also revealed no survival difference between real‐world patients receiving first‐line combination therapies and those with TKI monotherapy. Current guidelines recommend first‐line treatment with a single TKI (sunitinib or pazopanib) for patients with favorable IMDC risk, a pattern also seen in our data: patients with TKI monotherapy were more frequently of favorable risk compared to the other treatment groups (26% vs. 9%–17%).^1^, ^2^ Some patient groups may still benefit from first‐line treatment with a single TKI, particularly those in the real‐world who are typically excluded from RCTs, such as the elderly or those with comorbidities^44^ as also observed in our cohort. When stratified by IMDC risk, our results demonstrated no significant differences in OS among the three strategies. However, median OS was not yet reached for some groups, particularly for those at favorable risk. Only regarding PFS, patients at intermediate IMDC risk seem to benefit most from CPI‐TKI strategies.

*Third*, higher‐line treatment can also be a crucial factor for a patient's survival. Of all patients with first‐line TKI monotherapy included in this analysis who had already received second‐line treatment at the time of database cut (55%), the majority (77%) were treated with a CPI in second‐line, particularly CPI monotherapy. Therefore, the proportion of total CPI exposition in the TKI group (48%) was higher than that observed in most pivotal trials comparing CPI + TKI or CPI + CPI strategies in the sunitinib arm (23%–27%).^8^, ^11^, ^12^, ^13^ This may also help explain the favorable survival rates associated with TKI monotherapy, as demonstrated by our and other (retrospective) real‐world data.^42^, ^43^, ^45^

*Fourth*, it is important to note that the findings on the efficacy of an approved treatment for aRCC are largely based on the results of a single pivotal RCT.^3^ In the aforementioned Cochrane systematic review, pembrolizumab/axitinib—as the most commonly used CPI + TKI combination in our analysis—and ipilimumab/nivolumab were reported to probably improve OS across risk groups compared to sunitinib.^3^ While pembrolizumab plus lenvatinib may improve OS compared to sunitinib, no comparison data were available for avelumab plus axitinib and nivolumab plus cabozantinib.^3^ Therefore, further studies are required to directly compare these strategies head‐to‐head, rather than solely with sunitinib.^3^ Given that ongoing studies are not designed to compare different regimens, real‐world data represent an appropriate and valuable source to fill this gap of knowledge.^5^

Besides clinical effectiveness, HRQoL plays a decisive role in the management of aRCC. The correlation between QoL scores derived from the FKSI‐19 questionnaire and OS has been shown in real‐world patients with aRCC.^46^ Despite the survival benefits of combination strategies, they potentially represent increased toxicity when compared to monotherapy, which may negatively impact the QoL of patients.^47^ In our emulation, the median TTD of HRQoL did not greatly differ between the three first‐line strategies, neither overall nor stratified by patients' IMDC risk. In the pivotal trials, improvements in HRQoL were only demonstrated compared to sunitinib and were reported for the CPI + TKI strategies nivolumab/cabozantinib and pembrolizumab/lenvatinib, the CPI doublet ipilimumab/nivolumab, and for atezolizumab plus bevacizumab.^48^ Patients receiving first‐line pazopanib—as the most commonly used TKI in our analysis—reported a higher level of QoL than patients who were treated with sunitinib.^3^ Since the comparison of QoL endpoints across different trials is unreliable, future studies are warranted which should adopt best practices for the design, analysis, and reporting of PROs.^5^, ^48^

## 5 LIMITATIONS

Strengths of this work include the prospective, longitudinal design and the analysis of a large real‐world dataset. The emulation of a hypothetical target trial to adjust for confounders represents a significant methodological advantage that distinguishes this work from previous retrospective real‐world analyses in this context. Emulated targeted trials derived from real‐world data are, by design, constrained by the absence of randomization. The number and quality of potentially confounding variables are therefore crucial in determining interpretability. It cannot be excluded that confounders not documented in the data may have affected results. Although the set of included adjustment covariates was selected based on medical expertise and deemed sufficient, the possibility of unadjusted sources of confounding may lead to biased estimates. For the variables considered, the covariate balance was satisfactory for all covariates, thereby rendering the treatment groups comparable. Due to the observational design of CARAT, there are no specifications regarding the timing, frequency, or criteria of tumor assessment. Thus, rwPFS data should be considered as the best clinical approximation and might not be identical to PFS determined in clinical trials. A limitation inherent to all QoL assessments is the possibility of missing data, which is unlikely to be missing at random. Patients with deteriorating HRQoL are less likely to return questionnaires.

## 6 CONCLUSIONS

The findings of this analysis using a large real‐world dataset from the CARAT registry indicate no statistically significant differences in HRs for rwPFS and OS between the first‐line standards of care, CPI + CPI and CPI + TKI, when adjusted for a wide range of potential confounding variables. However, there is a trend toward improved survival with first‐line CPI + TKI compared to CPI + CPI, particularly for intermediate‐risk patients. First‐line treatment with TKI monotherapy may still be an option for selected patient populations in routine practice. Differences between real‐world patients and those selected for clinical trials, the choice of first‐line TKI, and subsequent treatment might explain differences in median PFS and OS in the present analysis compared to those reported from recently published phase III trials. While head‐to‐head randomized comparison trials are lacking, the emulation of a hypothetical target trial using real‐world data may help fill this knowledge gap and distinguishes this work from previous retrospective real‐world analyses. Further analyses, preferentially randomized clinical trials, are warranted to guide treatment decisions in clinical practice and improve patient care in aRCC, taking into account individual patients' preferences and needs.

## AUTHOR CONTRIBUTIONS

**Peter J. Goebell:** Conceptualization; investigation; resources; supervision; writing – review and editing. **Martin Bögemann:** Investigation; resources; writing – review and editing. **Arnd Nusch:** Investigation; writing – review and editing; resources. **Viktor Grünwald:** Conceptualization; investigation; resources; writing – review and editing. **Lothar Müller:** Conceptualization; investigation; writing – review and editing; resources. **Eyck von der Heyde:** Investigation; writing – review and editing; resources. **Uwe M. Martens:** Investigation; writing – review and editing; resources. **Carolin Lennartz:** Conceptualization; data curation; formal analysis; methodology; software; validation; writing – original draft. **Michaela Koska:** Conceptualization; data curation; project administration; visualization; writing – original draft. **Karin Potthoff:** Conceptualization; supervision; writing – review and editing. **Anja Kaiser‐Osterhues:** Conceptualization; methodology; visualization; writing – original draft. **Carsten Grüllich:** Conceptualization; investigation; resources; writing – review and editing. **Michael Staehler:** Conceptualization; investigation; writing – review and editing. **Martina Jänicke:** Conceptualization; methodology; supervision; visualization; writing – original draft. **Dominik Marschner:** Supervision; investigation; writing – review and editing; resources.

## FUNDING INFORMATION

CARAT is designed, managed, and analyzed by iOMEDICO and has received continuous financial support from MSD Sharp & Dohme GmbH and IPSEN PHARMA GmbH, as well as temporary support from EUSA Pharma GmbH and Eisai GmbH. All funders had no role in study design, data collection and analysis, interpretation of results, or decision to publish.

## CONFLICT OF INTEREST STATEMENT

Arnd Nusch, Eyck von der Heyde, Carolin Lennartz, Michaela Koska, Karin Potthoff, Anja Kaiser‐Osterhues, Carsten Grüllich, Martina Jänicke, and Dominik Marschner declare no conflict of interest concerning the topic of this publication. Peter J. Goebell: consulting or advisory roles with Bayer, Ipsen, and Novartis; honoraria for talks or speeches from Accord, AstraZeneca, Astellas, Apogepha, Bayer, BMS, Cepheid, Eisai, EUSA, Hexal, Ipsen, Janssen‐Cilag, Merck, MSD, Novartis, Pfizer, Recordati, Roche, Sandoz, Sanofi, and Takeda; participation at advisory boards and travel support from Accord, AstraZeneca, Astellas, Apogepha, Bayer, BMS, Cepheid, Eisai, EUSA, Hexal, Ipsen, Janssen‐Cilag, Merck, MSD, Novartis, Pfizer, Recordati, Roche, Sandoz, and Sanofi. Martin Bögemann: employment with Janssen; consulting or advisory roles with Bayer, Janssen‐Cilag, Astellas Pharma, AstraZeneca, MSD, Bristol‐Myers Squibb, Ipsen, Roche, Novartis, Merck, Sanofi, Eisai, and Gilead Sciences; travel, accommodations, and expenses covered by Janssen‐Cilag, Bayer, Amgen, and BMS GmbH & Co. KG; honoraria from Janssen‐Cilag, Astellas Pharma, Bayer/Vital, Sanofi/Aventis, MSD, Bristol‐Myers Squibb, Pfizer, Novartis, Ipsen, EUSA Pharma, Merck, Eisai, Amgen, AstraZeneca, Roche, and Advanced Accelerator Applications; research funding from Janssen‐Cilag and IPSEN. Volker Grünwald: employment with University Hospital Essen; consulting or advisory roles with Bristol‐Myers Squibb, Cureteq, Debiopharm Group, Eisai, Gilead Sciences, Ipsen, Janssen‐Cilag, MSD Oncology, Novartis, Oncorena, PCI Biotech, Pfizer, and Synthekine; travel, expenses covered by AstraZeneca, Ipsen, Janssen, Merck Serono, and Pfizer; stock ownership interests in AstraZeneca, Bicycle Therapeutics, Bristol‐Myers Squibb, Genmab, and MSD; honoraria from Advanced Accelerator Applications/Novartis, Amgen, Apogepha, Astellas Pharma, AstraZeneca, Bristol‐Myers Squibb, Eisai, Ipsen, Janssen‐Cilag, Merck Serono, MSD Oncology, Ono Pharmaceutical, and Pfizer; research funding from Amgen, Bicycle Therapeutics, Bristol‐Myers Squibb, Gilead Sciences, Ipsen, MSD Oncology, and Seagen. Lothar Müller: advisory role with Roche; travel, accommodations, and expenses covered by Octapharm and Pierre Fabre; honoraria from Octapharm. Uwe M. Martens: leadership role as founder of MOLIT Institute GmbH; stock and other ownership interests in Eli Lilly and Novo Nordisk; honoraria from Roche; consulting or advisory roles with MSD, Roche, BMS GmbH & Co. KG, Pfizer, Pierre Fabre, and Guardant Health; research funding from Dieter Schwarz Foundation; travel, accommodations, and expenses covered by Pierre Fabre, Roche, Ipsen, and Pfizer. Michael Staehler: consulting or advisory roles with Pfizer, Novartis, Ipsen, Exelixis, Eisai, Bristol‐Myers Squibb, EUSA Pharma, Merck Sharp & Dohme, EMD Serono, Apogepha, Oncorena, AstraZeneca, and Johnson & Johnson/Janssen; speakers' bureau roles with Pfizer, Novartis, Bristol‐Myers Squibb, Eisai, Ipsen, and EUSA Pharma; travel, accommodations, and expenses covered by Pfizer, Novartis, Bristol‐Myers Squibb, Eisai, Ipsen, EUSA Pharma, MSD Oncology, and EMD Serono; honoraria from Pfizer, Novartis, Roche, Ipsen, Bristol‐Myers Squibb, Exelixis, Bayer, EUSA Pharma, Incyte, Astellas Pharma, MSD Oncology, and EMD Serono; research funding from Pfizer, Roche/Genentech, Exelixis, Novartis, Bayer, Bristol‐Myers Squibb, and Eisai.

## ETHICS STATEMENT

The research platform CARAT was approved by the responsible ethics committee (Baden‐Württemberg, F‐2017‐085) and is registered at ClinicalTrials.gov (NCT03374267). Written informed consent was obtained from all patients.

## Supporting information


**Data S1.** Supporting Information.

## Data Availability

The data that support the findings of this study are available from the corresponding author upon reasonable request.

## References

[1] Ljungberg B , Bex A , Albiges L , et al. EAU guidelines on renal cell carninoma: update 2024. 2024. https://d56bochluxqnz.cloudfront.net/documents/pocket-guidelines/EAU-Pocket-on-Renal-Cell-Carcinoma-2024.pdf. Accessed 10 Jul 2024.

[2] Powles T , Albiges L , Bex A , et al. Renal cell carcinoma: ESMO clinical practice guideline for diagnosis, treatment and follow‐up. Ann Oncol. 2024;35:692‐706. 38788900 10.1016/j.annonc.2024.05.537

[3] Aldin A , Besiroglu B , Adams A , et al. First‐line therapy for adults with advanced renal cell carcinoma: a systematic review and network meta‐analysis. Cochrane Database Syst Rev. 2023;5:CD013798. 37146227 10.1002/14651858.CD013798.pub2 PMC10158799

[4] Quhal F , Mori K , Bruchbacher A , et al. First‐line immunotherapy‐based combinations for metastatic renal cell carcinoma: a systematic review and network meta‐analysis. Eur Urol Oncol. 2021;4:755‐765. 33757737 10.1016/j.euo.2021.03.001

[5] Navani V , Heng DYC . Treatment selection in first‐line metastatic renal cell carcinoma‐the contemporary treatment paradigm in the age of combination therapy: a review. JAMA Oncol. 2022;8:292‐299. 34792538 10.1001/jamaoncol.2021.4337

[6] Chen Y‐W , Wang L , Panian J , et al. Treatment landscape of renal cell carcinoma. Curr Treat Options Oncol. 2023;24:1889‐1916. 38153686 10.1007/s11864-023-01161-5 PMC10781877

[7] Motzer R , Alekseev B , Rha S‐Y , et al. Lenvatinib plus pembrolizumab or everolimus for advanced renal cell carcinoma. N Engl J Med. 2021;384:1289‐1300. 33616314 10.1056/NEJMoa2035716

[8] Choueiri TK , Powles T , Burotto M , et al. Nivolumab plus cabozantinib versus sunitinib for advanced renal‐cell carcinoma. N Engl J Med. 2021;384:829‐841. 33657295 10.1056/NEJMoa2026982 PMC8436591

[9] Albiges L , Tannir NM , Burotto M , et al. Nivolumab plus ipilimumab versus sunitinib for first‐line treatment of advanced renal cell carcinoma: extended 4‐year follow‐up of the phase III CheckMate 214 trial. ESMO Open. 2020;5:e001079. 33246931 10.1136/esmoopen-2020-001079 PMC7703447

[10] Powles T , Plimack ER , Soulières D , et al. Pembrolizumab plus axitinib versus sunitinib monotherapy as first‐line treatment of advanced renal cell carcinoma (KEYNOTE‐426): extended follow‐up from a randomised, open‐label, phase 3 trial. Lancet Oncol. 2020;21:1563‐1573. 33284113 10.1016/S1470-2045(20)30436-8

[11] Motzer RJ , Tannir NM , McDermott DF , et al. Nivolumab plus ipilimumab versus sunitinib in advanced renal‐cell carcinoma. N Engl J Med. 2018;378:1277‐1290. 29562145 10.1056/NEJMoa1712126 PMC5972549

[12] Rini BI , Plimack ER , Stus V , et al. Pembrolizumab plus axitinib versus sunitinib for advanced renal‐cell carcinoma. N Engl J Med. 2019;380:1116‐1127. 30779529 10.1056/NEJMoa1816714

[13] Motzer RJ , Penkov K , Haanen J , et al. Avelumab plus axitinib versus sunitinib for advanced renal‐cell carcinoma. N Engl J Med. 2019;380:1103‐1115. 30779531 10.1056/NEJMoa1816047 PMC6716603

[14] Haanen JBAG , Larkin J , Choueiri TK , et al. Extended follow‐up from JAVELIN renal 101: subgroup analysis of avelumab plus axitinib versus sunitinib by the International Metastatic Renal Cell Carcinoma Database Consortium risk group in patients with advanced renal cell carcinoma. ESMO Open. 2023;8:101210. 37104931 10.1016/j.esmoop.2023.101210 PMC10265611

[15] Bourlon MT , Escudier B , Burotto M , et al. Nivolumab plus cabozantinib (N+C) vs sunitinib (S) for previously untreated advanced renal cell carcinoma (aRCC): results from 55‐month follow‐up of the CheckMate 9ER trial. J Clin Oncol. 2024;42:362.

[16] Motzer RJ , Powles T , Burotto M , et al. Nivolumab plus cabozantinib versus sunitinib in first‐line treatment for advanced renal cell carcinoma (CheckMate 9ER): long‐term follow‐up results from an open‐label, randomised, phase 3 trial. Lancet Oncol. 2022;23:888‐898. 35688173 10.1016/S1470-2045(22)00290-X PMC10305087

[17] Heng DYC , Xie W , Regan MM , et al. Prognostic factors for overall survival in patients with metastatic renal cell carcinoma treated with vascular endothelial growth factor‐targeted agents: results from a large, multicenter study. J Clin Oncol. 2009;27:5794‐5799. 19826129 10.1200/JCO.2008.21.4809

[18] Bosma NA , Warkentin MT , Gan CL , et al. Efficacy and safety of first‐line systemic therapy for metastatic renal cell carcinoma: a systematic review and network meta‐analysis. Eur Urol Open Sci. 2022;37:14‐26. 35128482 10.1016/j.euros.2021.12.007 PMC8792068

[19] Santoni M , Buti S , Myint ZW , et al. Real‐world outcome of patients with advanced renal cell carcinoma and intermediate‐ or poor‐risk International Metastatic Renal Cell Carcinoma Database Consortium criteria treated by immune‐oncology combinations: differential effectiveness by risk group? Eur Urol Oncol. 2024;7:102‐111. 37481365 10.1016/j.euo.2023.07.003

[20] Stühler V , Herrmann L , Rausch S , Stenzl A , Bedke J . Real world data on IO‐based therapy for metastatic renal cell carcinoma. J Cancer Res Clin Oncol. 2022;149:3249‐3258. 35907009 10.1007/s00432-022-04173-0 PMC10314860

[21] Gustave Roussy . CARE1: a European study on first‐line treatment of metastatic kidney cancer [Internet]. Gustave Roussy. https://www.gustaveroussy.fr/en/care1-european-study-first-line-treatment-metastatic-kidney-cancer. Accessed 10 Jul 2024

[22] Goebell PJ , Staehler M , Müller L , et al. Changes in treatment reality and survival of patients with advanced clear cell renal cell carcinoma – analyses from the German clinical RCC‐registry. Clin Genitourin Cancer. 2018;16:e1101‐e1115. 30061035 10.1016/j.clgc.2018.06.006

[23] Goebell PJ , Müller L , Hübner A , et al. Body mass index as independent predictor of overall survival in patients with advanced renal cell carcinoma at start of systemic treatment—analyses from the German clinical RCC‐registry. Urol Oncol Semin Orig Investig. 2018;36:470.e1‐470.e9. 10.1016/j.urolonc.2018.07.007 30131294

[24] Staehler M , Goebell PJ , Müller L , et al. Rare patients in routine care: treatment and outcome in advanced papillary renal cell carcinoma in the prospective German clinical RCC‐registry. Int J Cancer. 2020;146:1307‐1315. 31498894 10.1002/ijc.32671 PMC7003963

[25] Marschner N , Staehler M , Müller L , et al. Survival of patients with advanced or metastatic renal cell carcinoma in routine practice differs from that in clinical trials‐analyses from the German clinical RCC registry. Clin Genitourin Cancer. 2017;15:e209‐e215. 27720164 10.1016/j.clgc.2016.08.022

[26] Kaplan EL , Meier P . Nonparametric estimation from incomplete observations. J Am Stat Assoc. 1958;53:457‐481.

[27] Boyne DJ , Brenner DR , Gupta A , et al. Head‐to‐head comparison of FOLFIRINOX versus gemcitabine plus nab‐paclitaxel in advanced pancreatic cancer: a target trial emulation using real‐world data. Ann Epidemiol. 2023;78:28‐34. 36563766 10.1016/j.annepidem.2022.12.005

[28] Cole SR , Hernan MA . Constructing inverse probability weights for marginal structural models. Am J Epidemiol. 2008;168:656‐664. 18682488 10.1093/aje/kwn164 PMC2732954

[29] Austin PC , Stuart EA . Moving towards best practice when using inverse probability of treatment weighting (IPTW) using the propensity score to estimate causal treatment effects in observational studies. Stat Med. 2015;34:3661‐3679. 26238958 10.1002/sim.6607 PMC4626409

[30] Zhang Z , Kim HJ , Lonjon G , Zhu Y . Balance diagnostics after propensity score matching. Ann Transl Med. 2019;7:16. 30788363 10.21037/atm.2018.12.10 PMC6351359

[31] Stuart EA , Lee BK , Leacy FP . Prognostic score‐based balance measures can be a useful diagnostic for propensity score methods in comparative effectiveness research. J Clin Epidemiol. 2013;66:S84‐S90.e1. 23849158 10.1016/j.jclinepi.2013.01.013 PMC3713509

[32] Royston P , Parmar MKB . The use of restricted mean survival time to estimate the treatment effect in randomized clinical trials when the proportional hazards assumption is in doubt. Stat Med. 2011;30:2409‐2421. 21611958 10.1002/sim.4274

[33] Butt Z , Peipert J , Webster K , Chen C , Cella D . General population norms for the functional assessment of cancer therapy‐kidney symptom index (FKSI): general population norms for the FKSI. Cancer. 2013;119:429‐437. 22778010 10.1002/cncr.27688 PMC3470751

[34] Cella D , Grünwald V , Escudier B , et al. Patient‐reported outcomes of patients with advanced renal cell carcinoma treated with nivolumab plus ipilimumab versus sunitinib (CheckMate 214): a randomised, phase 3 trial. Lancet Oncol. 2019;20:297‐310. 30658932 10.1016/S1470-2045(18)30778-2 PMC6701190

[35] Quan H , Li B , Couris CM , et al. Updating and validating the Charlson comorbidity index and score for risk adjustment in hospital discharge abstracts using data from 6 countries. Am J Epidemiol. 2011;173:676‐682. 21330339 10.1093/aje/kwq433

[36] Gan CL , Dudani S , Wells JC , et al. Outcomes of first‐line (1L) immuno‐oncology (IO) combination therapies in metastatic renal cell carcinoma (mRCC): results from the international mRCC database consortium (IMDC). J Clin Oncol. 2021;39:276.

[37] Zarrabi KK , Handorf E , Miron B , et al. Comparative effectiveness of front‐line ipilimumab and nivolumab or axitinib and pembrolizumab in metastatic clear cell renal cell carcinoma. Oncologist. 2023;28:157‐164. 36200791 10.1093/oncolo/oyac195 PMC9907035

[38] Chakiryan NH , Jiang DD , Gillis KA , et al. Real‐world survival outcomes associated with first‐line immunotherapy, targeted therapy, and combination therapy for metastatic clear cell renal cell carcinoma. JAMA Netw Open. 2021;4:e2111329. 34032854 10.1001/jamanetworkopen.2021.11329 PMC8150693

[39] Hoeh B , Schmucker P , Klümper N , et al. Comparison of first‐line anti‐PD‐1‐based combination therapies in metastatic renal‐cell carcinoma: real‐world experiences from a retrospective, multi‐institutional cohort. Urol Int. 2022;106:1150‐1157. 35158357 10.1159/000521661

[40] Heng DYC , Choueiri TK , Rini BI , et al. Outcomes of patients with metastatic renal cell carcinoma that do not meet eligibility criteria for clinical trials. Ann Oncol. 2014;25:149‐154. 24356626 10.1093/annonc/mdt492 PMC4155479

[41] Choueiri TK , Halabi S , Sanford BL , et al. Cabozantinib versus sunitinib As initial targeted therapy for patients with metastatic renal cell carcinoma of poor or intermediate risk: the alliance A031203 CABOSUN trial. J Clin Oncol. 2017;35:591‐597. 28199818 10.1200/JCO.2016.70.7398 PMC5455807

[42] Shah NJ , Sura SD , Shinde R , et al. Real‐world treatment patterns and clinical outcomes for metastatic renal cell carcinoma in the current treatment era. Eur Urol Open Sci. 2023;49:110‐118. 36874600 10.1016/j.euros.2022.12.015 PMC9974999

[43] Rizzo M , Pezzicoli G , Tibollo V , Premoli A , Quaglini S . Clinical outcome predictors for metastatic renal cell carcinoma: a retrospective multicenter real‐life case series. BMC Cancer. 2024;24:804. 38970009 10.1186/s12885-024-12572-4 PMC11225140

[44] Rossi E , Bersanelli M , Gelibter AJ , et al. Combination therapy in renal cell carcinoma: the best choice for every patient? Curr Oncol Rep. 2021;23:147. 34748099 10.1007/s11912-021-01140-9 PMC8575734

[45] Lai G‐S , Li J‐R , Wang S‐S , et al. Real world treatment sequences and outcomes for metastatic renal cell carcinoma. PLoS One. 2023;18:e0294039. 37992086 10.1371/journal.pone.0294039 PMC10664936

[46] Lendínez‐Cano G , Vilches‐Arenas Á , Congregado‐Ruíz B , Medina‐López R . Patient's self‐reported quality of life as a prognostic factor in metastatic renal cell carcinoma initially treated with TKI: nomogram proposal. World J Urol. 2024;42:267. 38678165 10.1007/s00345-024-04972-9

[47] Fontes‐Sousa M , Magalhães H , Oliveira A , et al. Reviewing treatment options for advanced renal cell carcinoma: is there still a place for tyrosine kinase inhibitor (TKI) monotherapy? Adv Ther. 2022;39:1107‐1125. 35025061 10.1007/s12325-021-02007-y PMC8756748

[48] Nolla K , Benjamin DJ , Cella D . Patient‐reported outcomes in metastatic renal cell carcinoma trials using combinations versus sunitinib as first‐line treatment. Nat Rev Urol. 2023;20:420‐433. 36928615 10.1038/s41585-023-00747-w

