# Supplementary material for: Head‐to‐head comparison of TKI and CPI first‐line treatment strategies in advanced renal cell carcinoma—Real‐world data from the German research platform CARAT
Source: Int J Cancer. 2025 Oct 29;158(6):1690–702. doi: 10.1002/ijc.70211 (PMC12811213; doi:10.1002/ijc.70211)
Supplement: Supplementary file 1 — Data S1. Supporting Information. [file IJC-158-1690-s001.pdf]

Head-to-head comparison of TKI and CPI first-line treatment strategies in advanced renal cell carcinoma – real-world data from the German research platform CARAT

Peter J. Goebell, Martin Bögemann, Arnd Nusch, Viktor Grünwald, Lothar Müller, Eyck von der Heyde, Uwe M. Martens, Carolin Lennartz, Michaela Koska, Karin Potthoff, Anja Kaiser-Osterhues, Carsten Grüllich, Michael Staehler, Martina Jänicke, Dominik Marschner and the CARAT Registry Group

Supplementary Material – Table of Contents

Supplementary Table 1. Specification and emulation of a target trial 2

Supplementary Table 2. Balance of baseline characteristics before and after weighting 3

Supplementary Figure 1. Covariate balance of IPTW 4

Supplementary Figure 2. Time to deterioration of health-related quality of life (adjusted, after IPTW) 5

Supplementary Table 1. Specification and emulation of a target trial

**Protocol item**	**Target trial**	**Emulation**
Eligibility criteria	▪ Diagnosis of advanced renal cell carcinoma<br>▪ Locally advanced, unresectable or metastatic disease<br>▪ Patients ≥18 years of age<br>▪ Prospective inclusion	▪ Diagnosis of advanced renal cell carcinoma<br>▪ Locally advanced, unresectable or metastatic disease<br>▪ Patients ≥18 years of age<br>▪ Prospective and retrospective inclusion until 12 weeks after start of treatment<br>▪ Patients with documented first-line treatment of either TKI+CPI or CPI+CPI or TKI monotherapy<br>▪ No withdrawal of informed consent<br>▪ No patients violating the CARAT inclusion criteria during the study
Treatment strategies (first-line)	1. TKI+CPI<br>2. CPI+CPI<br>3. TKI monotherapy	Same as target trial
Treatment assignment	Individuals were randomly assigned to a treatment strategy and were aware of their assignment	Individuals were classified to their treatment strategy compatible with their observed data
Outcome	▪ Progression<br>▪ Death from any cause<br>▪ Time-to-deterioration of health-related quality of life	Same as target trial
Follow-up	Follow-up started at time of first-line treatment assignment and ended at death, date of last contact, or date of data base cut (December 31, 2023), whichever occurred first	Same as target trial
Causal contrasts<br>Analysis plan	Intention-to-treat effect:<br>▪ Sample: all<br>▪ Analysis: Kaplan-Meier analysis, groups according to randomization<br><br>Per-protocol effect:<br>▪ Sample: only patients not violating the inclusion criteria and only receiving TKI+CPI, CPI+CPI or TKI monotherapy<br>▪ Analysis: Kaplan-Meier analysis, groups according to actual treatment	Per-protocol effect:<br>▪ Sample: same as target trial<br>▪ Analysis: Inverse probability of treatment Kaplan-Meier analysis, groups according to actual treatment

Adapted from and modified according to Boyne et al. 2023. *Note*: monitoring is less accurate in real-world treatment (e.g. no regular check for progression).
Abbreviations: CPI, checkpoint inhibitor; TKI, tyrosine kinase inhibitor.

Supplementary Table 2. Balance of baseline characteristics before and after weighting

Characteristic at start of first-line treatment	Original cohort				Weighted cohort[a]			
	CPI+TKI n= 447	CPI+CPI n= 257	TKI n= 166	SMD	CPI+TKI n= 446.68	CPI+CPI n= 256.55	TKI n= 166.03	SMD
Age in years, mean (StD)	68.08 (±10.30)	65.37 (±10.88)	72.61 (±10.73)	0.452	68.14 (±10.54)	68.24 (±10.88)	67.65 (±11.88)	0.035
Sex								
Female	33.8	32.7	35.5	0.040	34.1	33.4	33.0	0.015
Any comorbidity				0.203				0.046
Yes	87.7	85.6	94.6		88.5	89.1	90.6	
No	12.3	14.4	5.4		11.5	10.9	9.4	
CCI[b]				0.133				0.023
0	62.4	64.6	54.8		61.4	60.7	59.7	
≥1	37.6	35.4	45.2		38.6	39.3	40.3	
Histology				0.065				0.064
Clear cell ca	78.5	75.9	76.5		77.1	77.4	74.6	
Non-clear cell ca	21.3	24.1	23.5		22.8	22.6	25.4	
Missing	0.2	0.0	0.0		0.1	0.0	0.0	
Metastases at diagnosis				0.280				0.061
M0	38.5	33.1	43.4		37.8	38.2	38.7	
M1	47.9	54.1	36.1		47.4	46.7	46.5	
MX	13.0	12.8	20.5		14.4	15.1	14.8	
Missing	0.7	0.0	0.0		0.3	0.0	0.0	
Number of metastatic sites				0.065				0.016
0	6.5	4.7	6.6		5.8	5.6	5.8	
1	34.7	33.5	33.7		34.4	33.6	33.7	
≥2	58.8	61.9	59.6		59.8	60.9	60.4	
Selected metastatic locations[c] – not affected								
Liver	84.3	81.7	85.5	0.069	84.2	84.1	84.2	0.001
Lung	42.7	32.7	37.3	0.139	38.8	38.5	39.1	0.008
Bones	70.0	68.5	68.7	0.022	69.8	69.4	69.9	0.008
Brain	93.5	96.1	96.4	0.088	94.7	94.7	95.6	0.027
Pancreas	94.2	94.2	90.4	0.096	93.2	93.7	93.1	0.016
Clinical IMDC				0.408				0.043
Favorable risk	17.2	9.3	25.9		16.5	17.2	16.1	
Intermediate risk	50.3	47.9	39.2		47.4	47.0	49.1	
Poor risk	21.3	33.1	17.5		23.8	23.9	22.1	
Missing	11.2	9.7	17.5		12.3	11.9	12.7	

Note: Data are %, unless otherwise indicated. Some percentages might not add up to 100% due to rounding.

[a] For the weighted cohort, n refers to sum of weights; in order to increase readability, we have refrained from presenting the patient numbers (n) for the given characteristics.;

[b] Charlson comorbidity index (CCI) according to Quan et al., 2011;

[c] Multiple answers possible, i.e. patients can have more than mentioned metastases (e.g. bone and liver metastases); data collected from 8 weeks before to 4 weeks after start of treatment.

Abbreviations: ca, carcinoma; CCI, Charlson comorbidity index; CPI, checkpoint inhibitor; IMDC, International Metastatic Renal Cell Carcinoma Database Consortium; SMD, standardised mean difference; StD, standard deviation; TKI, tyrosine kinase inhibitor.

.

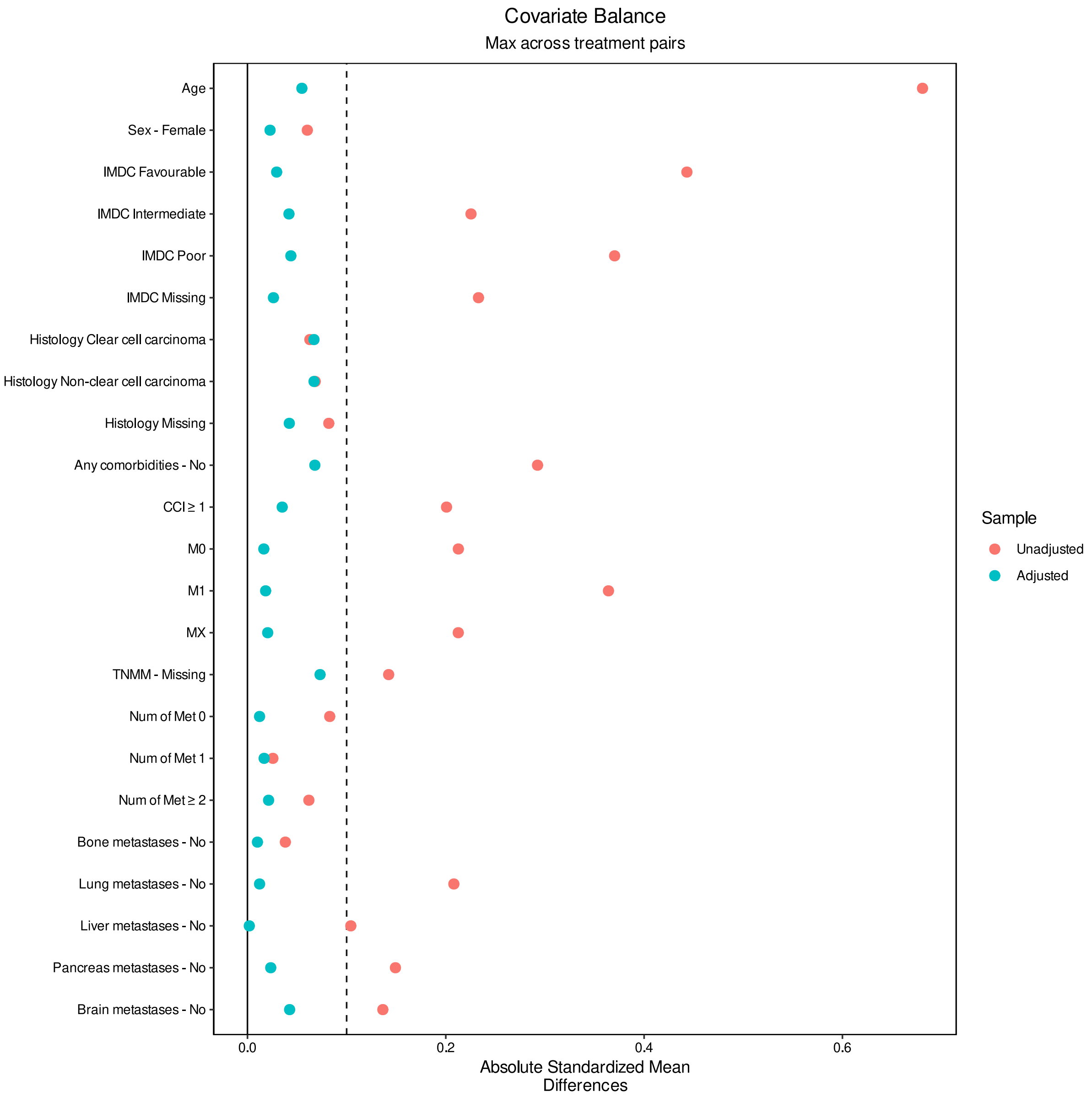

Supplementary Figure 1. Covariate balance of IPTW

Love plot summarizing the standardized mean differences before and after propensity score weighting (balance of individual covariates) for the total patient population. Balance is determined by an adjusted standardized mean difference <0.1, all covariates outside of this range could indicate imbalance. **Abbreviations:** CCI, Charlson comorbidity index; IMDC, International Metastatic Renal Cell Carcinoma Database Consortium; Met, metastasis/metastases.

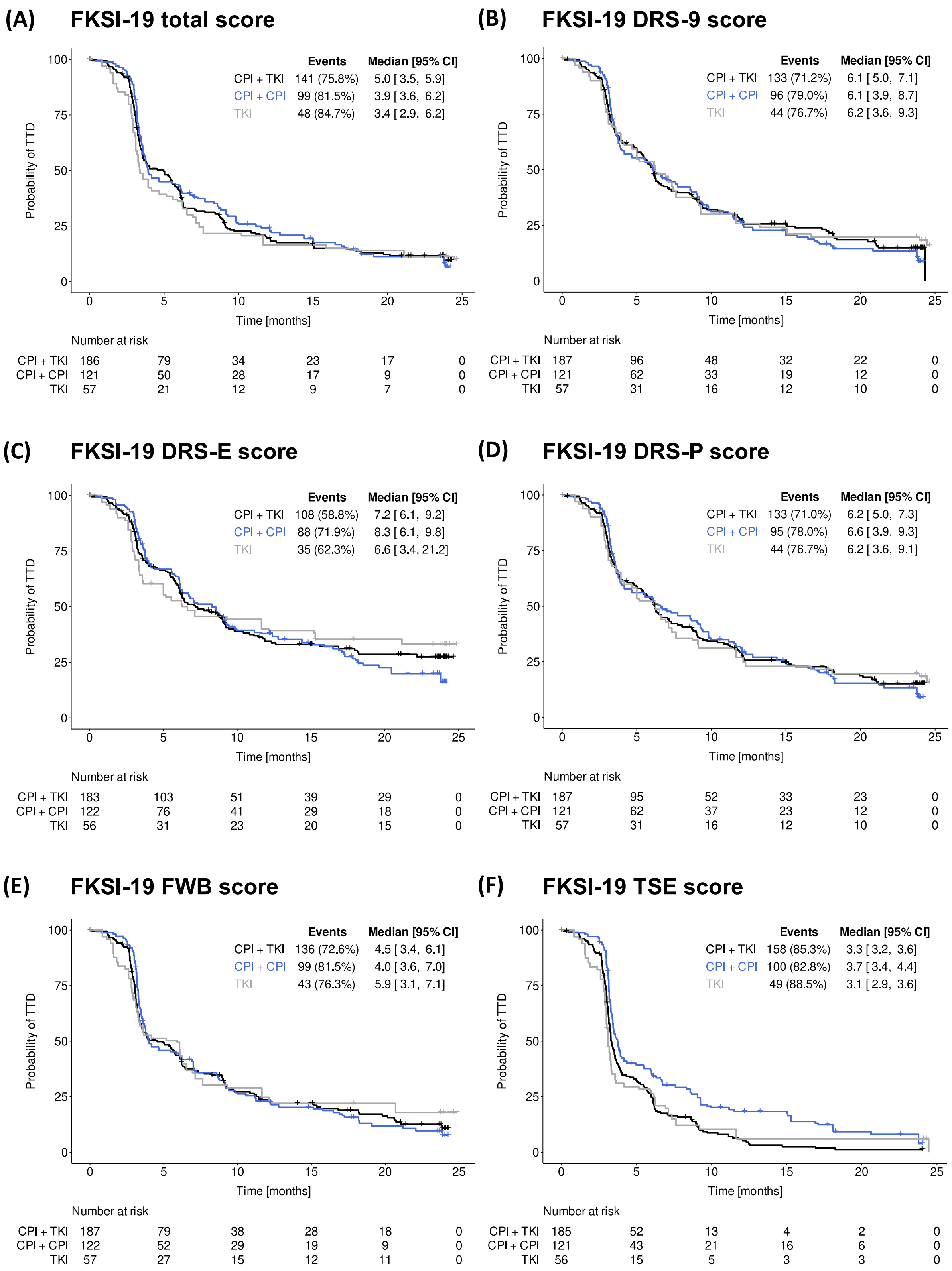

Supplementary Figure 2. Time to deterioration of health-related quality of life (adjusted, after IPTW)

TTD of HRQoL after weighting in patients receiving either CPI+TKI, CPI+CPI or TKI monotherapy for the FKSI-19 total scale **(A)**, the nine-item FKSI-19 disease-related symptoms scale **(B)**, the FKSI-19 disease-related symptoms-emotional subscale **(C),** the FKSI-19 disease-related symptoms-physical subscale **(D)**, the FKSI-19 functional well-being subscale **(E)** and the FKSI-19 treatment side effects subscale **(F)**. Numbers at risk refer to the sum of weights of the respective patients at risk for a given time point. **Abbreviations:** CI, confidence interval; CPI, checkpoint

inhibitor; DRS, disease-related symptoms; DRS-E, disease-related symptoms-emotional; DRS-P, disease-related symptoms-physical; FKSI-19, 19-item Functional Assessment of Cancer Therapy–Kidney Symptom Index; FWB, functional well-being; HRQoL, health-related quality of life; TKI, tyrosine kinase inhibitor; TSE, treatment side effects; TTD, time to deterioration.
